# Current Innovative Methods of Fetal pH Monitoring—A Brief Review

**DOI:** 10.3390/diagnostics12112675

**Published:** 2022-11-03

**Authors:** Roxana-Elena Bohiltea, Bianca Margareta Mihai, Ioniță Ducu, Ana-Maria Cioca, Alexia-Teodora Bohiltea, Ana-Maria Iordache, Stefan-Marian Iordache, Cristiana Eugenia Ana Grigorescu, Silviu Marinescu

**Affiliations:** 1 Department of Obstetrics and Gynecology, “Carol Davila” University of Medicine and Pharmacy Bucharest, 37 Dionisie Lupu, 020021 Bucharest, Romania; 2 Department of Obstetrics, Gynecology and Neonatology, Filantropia Hospital, 11-13 Ion Mihalache Blv., Sector 1, 011171 Bucharest, Romania; 3 Memorial Life Hospital, Grivitei Route, No. 365, 010719 Bucharest, Romania; 4 Faculty of Medicine, “Carol Davila” University of Medicine and Pharmacy Bucharest, 37 Dionisie Lupu, 020021 Bucharest, Romania; 5 École Hôtelière de Lausanne, 1000 Lausanne, Switzerland; 6 Optospintronics Department, National Institute for Research and Development in Optoelectronics-INOE 2000, 409 Atomistilor, 077125 Magurele, Romania; 7 Department 11-Plastic and Reconstructive Surgery, Pediatric Surgery, University of Medicine and Pharmacy “Carol Davila”, Eroii Sanitari Bvd., No. 8, Sector 5, 050471 Bucharest, Romania; 8 Discipline of Plastic Surgery and Reconstructive Microsurgery, Emergency Clinical Hospital “Bagdasar-Arseni”, Berceni Street, No. 12, Sector 4, 041915 Bucharest, Romania

**Keywords:** pH, fetal and neonatal monitoring, materno-fetal, fetal scalp pH sampling, electrochemical sensors, cardiotocografic monitoring, ultrasound monitoring, spectral monitoring of oxygen saturation

## Abstract

In this study, we explore the “why?”, and “how?”, monitoring the pH of the fetal scalp is used, and show its limitations. In addition, we review the development of new devices based on the modern physics and nanomaterials serving this topic. Most of the works we found in our search have focused on improving the prognostic of fetal heart rate monitoring, because it is the “golden standard” in determining fetal distress. Although the best-known screening method, it can only provide limited information about the actual status of the fetus. The best predictive assessment, with the highest reproducibility, states that a normal fetal heart rate is indicative of a healthy baby. However, its excellent sensitivity is much reduced when identifying the actual “distress”. This is when second-line monitoring methods come into play to guide the diagnostics and direct the obstetrician towards an action plan. Although a historic method, fetal scalp pH sampling is still under review as to its efficiency and place in the current obstetrics. Continuous surveillance of the fetal parameters is important, especially for the fetuses undergoing intrauterine growth restricted (IUGR). Since fetal scalp blood sampling is still under research and is a randomized controlled trial, which compares the relevance of pH and lactates to the obstetrical situation, the maternal-fetal medicine could greatly benefit from the introduction of engineered nanomedicines to the field.

## 1. Introduction

Perinatal mortality is at its lowest [1] due to a series of factors, among which: Superior antenatal, and intrapartum, care, periodic ultrasound monitoring, and assessment of the fetus, placenta, and regular fetal heart rate monitoring. Fetal heart rate (performed either through auscultation or electronically) is essential in the assessment and prediction of fetal oxygenation and acid-base status [2], allowing the clinician to evaluate the status of the fetus before deciding on the course of action. Recently, electronic fetal monitoring has become a hot topic in obstetrics, due to the new advances in material science that could be implemented in the field of materno-fetal medicine. In the light of a major increase in the incidence of primary cesarean births [3] scientists have searched methods to reduce its occurrence by adding the intrapartum fetal monitoring to steer towards surgical intervention only the cases showing complications. A recent estimate suggests that up to 75%^ cesarean sections were performed unnecessary [4]). Among those complications that require emergency intervention are fetal hypoxia and fetal distress. 

Intrapartum care aims to identify the fetuses in distress (compromised) via electronic fetal monitoring (EFM) as soon as possible. However, EFM cannot satisfactorily assess what kind of distress the fetus goes through (e.g., The incidence of cerebral palsy was not reduced since the introduction of EFM as a screening method) [5]. Along with cerebral palsy, acidemia in fetuses and newborns is difficult to diagnose. The acid-base status can be estimated/predicted by fetal heart rate (FHR), but not actively determined. Thus, second-line methods for fetal monitoring come into play. Since fetal acidosis is one of the major symptoms for birth hypoxia, the biochemical parameters, which need to be evaluated are pH, base deficit (concentration of bicarbonate ion) and lactate measurement. A recent study [6] focused on connecting the continuous EFM and arterial cord gas with clinical data, including pH, but the success was limited: the baseline and accelerations were correlated with normal pH (normal EFM features correlated with normal pH in the baby). The same study also determined that 149 out of the 8580 women undergoing labor had newborns with acidemia. Another study compared the cardiotocography with intermittent auscultation and fetal scalp blood and concluded that fetal blood sampling would decrease the cesarean rate associated with the use of continuous cardiotocography because of accurate identification of distress in fetuses [5,7].

Another means to determine pH is to measure the level of lactate by using test-strips. The test is simple, requires a low volume of blood (≈5 µL) and gives a rapid response (about 15 s). Nordstrom et al. [8], Westgren et al. [9] and Martin et al. [10] found that lactate is more effective than pH measurement for the assessment of metabolic acidemia. These studies found that scalp pH and scalp lactates are significantly correlated between them and with the umbilical artery lactate measurement. In terms of effectiveness of identification of perinatal hypoxia, lactate analysis seems to be equally effective as pH analysis. Another method for assessing the well-being of the unborn child is oximetry [11,12,13]. A 1998 study [14] showed that fetal arterial oxygen saturation data correlates significantly with scalp pH values, and together have a predictive value of fetal outcome. Not to mention that even laser Doppler flowmetry was used to evaluate the relationship between the variations in scalp blood flow and transcutaneous pO_2_ [15].

Together with fetal scalp sampling, lactate determination is used infrequently in the United States, but remains a common practice in other countries [16]. By performing a search in three digital libraries, we found few articles that compared the results from the cardiotocography with fetal scalp blood. In Romania, for fetal distress, the assessment of the fetal scalp pH is recommended, together with fetal cardiotocographic monitoring [17,18]. 

A very interesting controversy surrounding the usefulness of fetal scalp blood sampling [19,20] is left opened since 2014. The discussion has brought into light that although recommendations are given in the guidelines for the use of fetal scalp pH, there is scarce evidence of its efficiency because it was a technique performed decades ago, when fetal monitors were inexistent, and it should be removed from modern obstetrics and evidence-based medicine. However, Dr. Steer puts it straight: “the only way to assess whether a significant acidosis is developing is to measure the pH” [20]. In this study, we explore the “why?” and “how?” fetal scalp pH monitoring is used, which shows its limitations, and reviews the development of new devices based on the modern physics and nanomaterials. 

## 2. Materials and Methods

In order to obtain accurate scientific information and achieve a comprehensive understanding of the way pH scalp blood measurement works as an adjunctive or ‘second-line’ technique, we performed multiple ScienceDirect, Sage, and PubMed search queries using various site-specific keywords, such as: “fetal scalp pH”, “fetal blood analysis” and “Fetal tissue pH monitoring” (Appendix A). As a result, we identified more than 5000 short communications, research articles, abstracts, meeting reports or literature reviews, regarding various methods of prognostic, the level of pH of newborn during intrapartum. The inclusion criteria were: short communication, clinical opinion, research articles and reviews articles discussing fetal scalp pH and fetal blood monitoring. We included papers discussing both low risk and high risk, term and preterm pregnancies, with access to fetal blood sampling. The exclusion criteria were: type I duplicates (duplicates among different databases, as stated by [21]), abstract papers and meeting reports, studies dealing with other fetal monitoring without data about fetal blood sampling.

**Figure 1:**
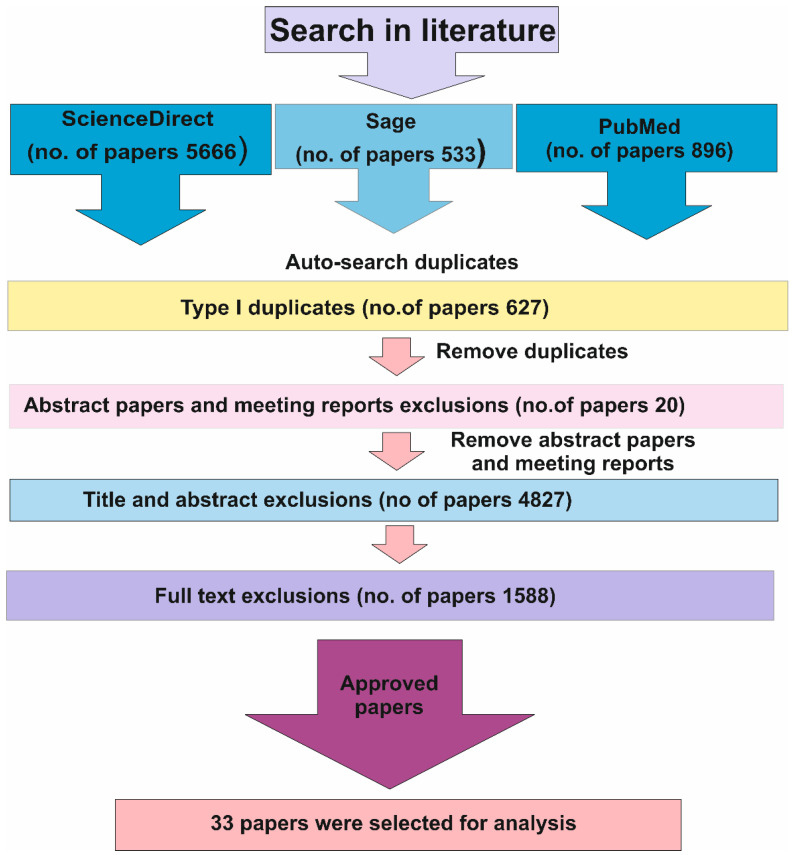
Study selection process.

There were only few methods of actively determining and monitoring. However, that may have been defined at that specific point in time, dating back as far as year 1983 [22]. After excluding title, abstract and full text, where studies unrelated to our research question were withdrawn from the review process, 33 papers were left for examination. The search strategy is based on Reference [23] and summarized in Figure 1.

## 3. Results and Discussions

Fetal acidemia is a rare occurrence, particularly during a scheduled cesarean section. A recent paper [24] found that out of 2081 planned cesarean births, 252 newborns had fetal acidemia at the time of delivery. A 12.1% fetal acidemia in a 10-year period represents a high incidence for this type of pregnancy risk and correct identification of acidemic neonates allows neonatal intensive care units to perform additional interventions, in order to decrease long-term adverse outcomes. The study found that fetuses in breech position, fetusesof women with obesity, and gestational diabetes mellitus are more prone to acidemia than fetuses in cephalic presentation and those born to women with a normal BMI (body mass index). Other identified risks included intraoperative maternal hypotension, use of vasopressor therapy, and prolonged time intervals between anesthesia and skin incision to delivery, which were positively correlated with fetal acidemia. An important finding is that fetal heart rate monitoring showed no correlation to the incidence of acidemia in fetuses. No matter what type of acidemia they have, fetuses with pH < 7.2 in the umbilical artery (pH was tested at the time of delivery) have an increased risk of adverse neonatal outcome. Intubation and respiratory distress are the most common and severe risks for the composite adverse neonatal outcome, compared to encephalopathy, therapeutic hypothermia, and seizures, which showed a low correlation. The authors calculated that 34% of the cases of acidemia had, in fact, mixed acidemia produced by hypoxia. This is a strange find because mixed and metabolic acidemia should not appear in scheduled cesarean delivery. The strength of this study was that it included only full term, perfectly healthy fetuses whose health was monitored during development. Still, 12% of those newborns were diagnosed with acidemia and 8.5% of those neonates had associated morbidities. 

Similarly, another recent study published by Frenken et al. [25] connected the frequency of uterine contractions with the fetal scalp pH. The study was a retrospective one, spanned over 5 years, between 2015 and 2020, and included 765 women with abnormal fetal heart rate. Their results supported those of Bligard et al. [24] showing that 18% (138) fetuses had hypoxia at birth. Uterine tachysystole was associated with fetal hypoxia, but the difference between contraction frequencies for women with a hypoxic fetus (4.4 contractions/10 min) vs. women without a hypoxic fetus (4 contractions/10 min) is relatively low. The authors concluded that the increase in the number of uterine contraction frequency prior is associated with fetal hypoxia, the highest risk being observed for women with more than 6 contractions/10 min. Frenkens’ study adds to Simpson et al. [26] because it verified that fetal oxygen saturation decreased with increasing contraction frequency no matter if the pregnant women had normal fetal heart rate (as in the case of Simpson et al.) or abnormal fetal heart rates (Frenkens’ case).

A special case is the fetuses diagnosed with congenital heart diseases. For these babies, the sensitivity and sensibility of the electronic heart rate monitor as well as blood gas analysis are lower than usual. A recent study performed by Feduniw et al. [27] showed that the rate of emergency cesarean delivery was three times higher for the fetuses with a prenatal diagnosis of congenital heart disease than for high-risk and low-risk pregnancies without this diagnosis. The authors concluded that acidemia did not play a role in the Apgar scoring of these newborns (they usually scored ≤ 7) and it could not be predicted by the intrapartum cardiotocography. This study comes as added value to a large systematic review published by Alfirevic et al. [28] who concluded that continuous cardiotocography is responsible for the increase in the number of caesarean sections, and the fetal outcome (e.g., seizures, etc.) was not influenced by results of the fetal blood sampling. 

### 3.1. Mechanism of Acidosis in Fetus (Why)

The oxygen reaches the fetus via maternal lungs → heart→ abdominal aorta →iliac artery → uterine artery → placenta → umbilical cord, with a final saturation in fetal hemoglobin of about 40–50% [5,29] During labor, because of hyperventilation, the maternal organism undergoes mild respiratory alkalosis shown by pH values of 7.5–7.6 in the antecubital vein. As the labor continues into delivery, the respiratory alkalosis is followed by mild metabolic acidosis due to the accumulation of fixed acids and, in particular, accumulation of lactic acid produced by a deficit in circulation. For a normal fetus, this means that the pH values decrease from 7.3 in early labor to 7.25 at delivery. Lower values indicate acidosis and distress: (1) a difference of 0.10 pH units between the maternal-fetal bloods is normal; (2) differences between 0.15–0.19 signify a pre-acidotic state and (3) differences ˃0.2 units of pH are a clear indication of acidosis [30].

There are two types of acidosis: (1) respiratory acidosis, which has no neurological long-term consequences for the newborn (pure respiratory acidosis occurs in the minutes before birth and is resolved by expelling the accumulated CO_2_ amount in the first breaths [5]) and is produced by the reaction of CO_2_ with water resulting in protons (H^+^) and bicarbonate ions; (2) metabolic acidosis, which is produced by anaerobic glycolysis of glucose into pyruvate, and the latter into lactate and protons, thus decreasing the pH. The metabolic acidosis is responsible for organ damages [5,31]. The consequences (either short- or long-term) depend on the severity of acidemia and the duration: acute acidemia last for hours while chronic acidemia spreads over days [32]. Blood sampling could steer the diagnosis of the type of fetal acidosis because if a high level of pCO_2_ is present, that means it is a respiratory acidosis, which in turn means an acute acidemia; if increased levels of lactic acid are present in the blood, that means metabolic acidosis, which indicates a chronic acidemia. An interesting point has been raised by Bobrow and Soothill [32] that even if severe hypoxia and severe acidosis occur at the time of delivery, the compensatory mechanisms kick-in trying to mitigate the problem and assure the survival of the baby if delivery happens shortly. Thus, it is important to have a real-time analysis of the pH of the blood in order to reduce the time interval between labor and delivery.

Moreover, there are other causes for hypoxic stress, such as compression of the umbilical cord, or utero-placental insufficiency. While the compression of the umbilical cord can be spontaneously repaired, the utero-placental insufficiency is difficult to restore. There are three types of placental insufficiency produced by three distinct mechanisms: (1) structural placental insufficiency—Produced by poor trophoblastic invasion into maternal spiral arterioles leading to the formation of small placental pools, which are insufficient to meet fetal oxygen requirements; (2) functional placental insufficiency—The structure of the villi and placental pools is normal, but abuse of specific drugs (e.g., oxytocin) may lead to a decrease in the placental pools; (3) relative utero-placental insufficiency (RUPI)—Everything is normal in terms of structure and functionality, but the metabolic demands of the fetus cannot be met by the oxygenated placental pools [33]. 

Contractions cause a reduction in the oxygen supply and accumulation of carbon dioxide, while uterine relaxation allows “flooding” of fresh oxygenated blood and release of carbon dioxide, together with other excretory products. In fetal inflammatory response syndrome produced by an ascending infection, if uterine contractions are allowed to continue, metabolic acidosis is initiated and causes fetal multi-organ damage.

If fetal hypoxia occurs, it is associated with mixed acidosis: the interference of diffusion of oxygen in the placenta perturbs the anaerobic glycolytic pathway, which leads to an accumulation of lactic acid in the amniotic fluid [30]. Acting as defense, the bicarbonate in the fluid tries to eliminate the lactic acid by converting it to CO_2_. This leads to a decrease in the bicarbonate ion and an increase in the partial pressure of CO_2_, translated into a decrease in the pH of fetal plasma. The initial causes responsible for the fetal hypoxic state will also impede the normal rapid transfer of the CO_2_ from the fetal circulation to the maternal circulation. The transplacental transfer between mother and fetus occurs rapidly for CO_2_ (it is lipid soluble) and slower for bicarbonate ion (charged molecule). This implies that if the mother has an accumulation of CO_2,_ it will be rapidly transmitted to the fetus. Similarly, if the maternal hyperventilation decreases the CO_2_ and increases the pH, these changes are transmitted rapidly to the fetus. However, if the cause for the hypoxia is related to the fetus (cord compression or placental dysfunction) the changes seen in the maternal organism could be altered (diluted) because of the size differences. 

Special problems are risen by maternal metabolic syndromes: if the acidosis (or alkalosis) is a maternal metabolic syndrome, it is transmitted to the fetus over a prolonged period, but it could not be reflected in the pH because the maternal organism would compensate a low bicarbonate ion concentration by lowering the CO_2_; this decrease, in turn, is transmitted to the fetus and CO_2_ will move from the fetus to the maternal circulation. Since the bicarbonate ion crosses slower than CO_2,_ the placental boundary, the fetal pH will increase. In this case, maternal acidosis will camouflage fetal acidosis. 

### 3.2. Methods of Assessment (How)

The method of fetal scalp blood sampling was first introduced in 1961, in Germany [30] and extensively studied in the 1970s [29]. It involved placing the pregnant woman in a lateral position and with the aid of an amnioscope, which protects the exposed area of the fetal scalp, 0.25 mL fetal blood was drawn from the scalp into a heparin-containing glass vial. Prior to blood drawn, the area was sterilized by evaporating ethyl chloride.

Several disadvantages have been identified: the patient needs to undergo at least 3–4 cm dilation and has the membranes ruptured, the medical personnel performing the technique needs to be extremely skilled, the need of serial pH determinations and unpredictability of the results should the tests be poorly done [29].

Some of these disadvantages were overcome by the miniature glass pH electrode [34]. This electrode is able to give out a continuous tissue pH reading provided by the pH-sensitive glass tip [34] which is inserted into the fetal scalp. The functioning principle of this pH meter is that it records the difference in the electrochemical potential between two solutions of different pH separated by a glass membrane. However, lacerations occurred due to head rotation during delivery and displacement of the electrode. A major advantage of the tissue pH electrode is that it does not require bleeding and can be applied without previous silicone spraying of the site, because it is inserted 3 mm in the scalp tissue [35]. A variation of the tissue electrode is the double helix spiral electrode [36]. This electrode was designed to record fetal ECG signal and to hold into place the tissue pH monitoring probe.

O’Dowd et al. [37] developed their own scalp pH probe after using a commercially available pH electrode from Kontron, Basel, Switzerland as reference. The Kontron probe requires that the electrode tip is introduced into the scalp skin through a 2 mm deep incision and has problems involving movements due to labor and contamination with amniotic fluid. The authors fabricated a pH ion-selective membrane based on tri-n-dodecylamine for their fetal probe, which showed excellent results, but it was tested only in vitro.

Nowadays, fetal blood pH requires only 35 µL of blood [38] and is analyzed off-site with Rapidlab 248^®^. Although an unpopular procedure (for this particular case it was performed for 5.4% of pregnant women undergoing vaginal delivery), it has strict clinical guidelines: NORMAL = pH ˃ 7.25; ATTENTION pH = 7.21–7.25 (if the pH is in this range, another sample is required to be tested in 30 min), DANGER pH = 7.16–7.20 (retest and begin delivery) and CRITICAL pH < 7.15 (immediate delivery). 

Even if studies have shown that fetuses monitored via fetal blood sampling were admitted less often to the NICU, most of the medical professionals prefer to use non-invasive techniques such as electronic heart rate monitoring [7], or intrapartum fetal pulse oximetry [13]. Although effortless and simple, those techniques have low sensitivity and sensibility and provide minimal information about pH values, values that can be obtained via direct measurements of the blood [39].

But fetal blood sampling (scalp and/or umbilical) could offer much more information than just pH. Other parameters such as base excess or bicarbonate (which are not measured but are calculated from the pH and pCO_2_ [32]), lactate measurement and hemoglobin are good candidates for fetal monitoring [28]. The cut-off values for these parameters are the following: (a) For pCO_2_, it varies as a function of labor stage: in early stage of the labor, the partial pressure is between 43–57 mmHg, while for the active phase with full dilatation it ranges from 36–60 mmHg [40]. Respiratory acidosis is installed at pCO_2_ ≥ 75 mmHg [5]; (b) base deficit: in early labor, the normal values are between 0.2–0.4 mEq/L, rising to 2–3.3 mEq/L in active labor with full dilatation [41]. In the case of severe metabolic acidosis, the level of the bicarbonate rises above 12mmol/L [5]; (c) lactate levels increase in severe metabolic acidosis above 10 mmol/L and an indication for cesarean delivery comes for lactate levels above 4.8 mmol/L [5]; (d) hemoglobin values below 40 g/L could indicate the presence of certain fetal conditions (e.g., rhesus disease anemia, infections, α-thalassemia or hemorrhages) [32]. A summary of the papers we included in this review is presented in Table 1. 

### 3.3. Perspectives (Mitigation of Disadvantages)

An actual upgrade of the technique was developed by Uchida et al. [25] in 2016. Since traditional fetal scalp blood sampling is disregarded by obstetricians due to its invasiveness, the researchers created a miniaturized, portable oximeter (fabricated using a flexible substrate with two LEDs, and two photodiodes adhered to a black rubber sheet) with the sensor attached to the examiners’ index finger. This way, they are using fetal head tissue oxygen saturation and correlate it with the pH of umbilical cord artery blood. The sensor showed promising results on mice. The spectral characteristics of hemoglobin and deoxyhemoglobin (assessed via the two LEDs) could be used to provide an estimation of pO_2_ level and thus, can be used as an indirect predictor for the pH in the fetal scalp blood. 

The clinical value of fetal scalp pH sampling is debatable. The technique was developed at a time when fetal heartbeat monitoring was performed by auscultation and was an objective alternative to an otherwise subjective measurement (the auscultation depended on the proficiency of each obstetrician). Some authors [33] believe that the skin on the fetal scalp is not a good measure of the oxygenation status of the fetus due to catecholamine induced vasoconstriction in early stages of fetal stress response. That is why this technique was abandoned in the USA. However, recent studies in Finland and Sweden show that fetal blood sampling can reduce the number of unnecessary caesarian sections by actively determining the presence of acidemia [24,25]

The fate of fetal scalp pH sampling is strongly connected to the added value it brings to the continuous fetal heart rate monitoring and to the decrease in the frequency of delivery by cesarean section, [30,48]. Since the 1980s, the problems of fetal hypoxia during labor were debated in journal articles and international forums [37]. Abnormal fetal heart rate patterns are not correlated with the acid-base status of the fetus; this correlation can only be done by an effective sampling of the fetal scalp blood pH. Therefore, alternatives to the sampling method are searched for. 

One such alternative is represented by the in vivo implanted electrochemical multiparametric microsensors. Dulay et al. [49] developed a micro-implantable array of pH and pO_2_ sensors for monitoring hypoxia in tissues. The experimental model was comprised of the sensor for pH (based on polypyrrole coated Pt working electrode) and the sensor for pO_2_ (based on Pt-Nafion working electrode), which were implanted in vivo in the iliac artery, and in the femur muscle, and were connected to a portable multipotentiostate, which recorded the chronoamperometric response of the sensors in hypoxia conditions. 

This microsensors technology creates a new generation of implantable/technology at the fingertip diagnostic tools for fetal surveillance. The most important challenges that need to be overcome by fetal scalp blood sampling are the technical (wires coming out of the cervix are neither comfortable to the patient nor to the doctor), invasiveness (blood sampling involves inserting a pH meter to the blood capillaries) and logistics challenges (with the development of modern electronics, the results could be transmitted directly to the medic via internet-of-things—IoT) [50]. Two emerging monitoring systems are currently tested to improve the labor and birthing experience: (1) Monica Novii^®^ and (2) Moyo. Both of them are fetal heart monitors that allow women to be mobile during labor and were created for high body mass index (BMI) patients. 

Spectral sensors are at the forefront of the development of medical diagnostic devices because they are resistant to electromagnetic interference, they are not subjected to electrical short-circuits and, mostly importantly, they are capable of non-invasive examination. Moreover, if miniaturization is possible, they can be cheap. Since they are capable of recognizing unique spectral signatures of the biochemical compounds involved in the physiology of the human body, they can be used in the comfort of the home and the development of telemedicine in the 21st century [52].

Continuous surveillance of the fetal parameters is important, especially for the fetuses undergoing intrauterine growth restricted (IUGR), who have limited oxygen reserves [53]. In this respect, the field of engineered nanomedicines, which include polymeric nanoparticles, liposomes, dendrimers, peptide-drug conjugates or viral vectors that have the advantage of being easily modifiable, targetable and miniaturized in terms of size, shape and surface chemistry to the needs of the perinatal care. Since fetal scalp blood sampling is still under research and randomized controlled trial, which compares the relevance of pH and lactates to the obstetrical situation, the maternal-fetal medicine could greatly benefit from the introduction of engineered nanomedicines to the field [25,51].

## 4. Conclusions

Most of the studies we have found focused on improving the prognostic of fetal heart rate monitoring because it is the “golden standard” for determining fetal distress. Although it is the best screening method, it can only provide limited information about the actual status of the fetus (the most predictive assessment, with the highest reproducibility, is that a normal fetal heart rate is indicative of a healthy baby). However, its excellent sensitivity is substantially reduced when identifying the actual “distress”. This is when second-line monitoring methods come into play to guide the diagnostics and allow the obstetrician towards an action plan. Although a historic method, fetal scalp pH sampling is still under review as to its efficiency and place in the current obstetrics. Engineered nanomedicine and modern electronics combined with IoT could assure its survival in today’s society. As Dr. Steer puts it: the main value of the fetal blood sampling is not in detecting acidosis but in demonstrating that when hypoxia is suspected, the fetus is not yet metabolically acidotic if a persisting relatively normal pH is shown; therefore, he does not need urgent delivery. Our “take-home message” is that all fetal monitoring systems are just a tool that cannot prevent injury but can alert if something is wrong. Injury is prevented only by the interdisciplinary obstetric team.

## Figures and Tables

**Table 1:** Summary of the studies selected.

First Author, Year of Publication	Country	Study Type	No. of Patients	Procedure Performed *	Measured Outcomes **	Main Findings	Ref.
Williams, 2003	USA	Retrospective study	488	electronic fetal monitoring and umbilical artery cord gas analysis	neonatal acidemia	Minimal/absent variability in the electronic fetal monitoring for at least 1 h and/or late deceleration are a sign of acidemia	[42]
Haverkamp, 1979	USA	Controlled prospective study	690	auscultation, electronic fetal monitoring, and electronic monitoring with scalp blood sampling	Apgar scores, cord blood gases, neonatal death, neonatal morbidity, nursery course	The rate of cesarean section was the highest (18%) for the electronically monitored group	[7]
Nordstrom, 1994	Sweden	Experimental study	66	lactate concentration in fetal scalp blood was assessed with a new test strip method	Lactate concentrations and pH values	There were no significant differences between lactate concentrations and pH values in early compared to late first stage of labor.	[8]
Westgren, 1998	Sweden	Randomized experimental study	341	Fetal scalp blood sampling following modified heart rate patterns	Lactate measurement and pH analysis	The procedure for lactate measurement was more successful that the one for pH analysis but both lactate and pH in fetal scalp blood can predict perinatal outcome	[9]
Clark, 1985	USA	Clinical opinion	-	Fetal heart monitoring vs. fetal scalp blood sampling	Apgar score, fetal compromise	Better prepared medical professionals could predict fetal distress from the fetal heart rate patterns	[43]
Valverde, 2011	Spain	Randomized experimental study	180	pulse oximetry vs. fetal electrocardiography	rate of cesarean delivery, indications for operative delivery due to NRFHR, and repercussions on the newborn’s acid–base status	Fetal electrocardiography reduced the rate of emergency cesarean delivery	[11]
Goffinet, 1997	France	prospective multicenter observational study	174	Fetal pulse oximetry and fetal blood analysis	fetal oxygen saturation and fetal blood analysis (pH, pO_2_)	Low fetal oxygen saturation (<30%) leads to poor neonatal condition	[12]
Seelback-Gobel, 1999	Germany	Multicenter retrospective study	400	Fetal pulse oximetry	Fetal acidosis	Arterial oxygen saturation less than 30% is confirmed as the threshold for fetal compromise during labor. Acidosis is triggered by the duration of hypoxemia.	[13]
Kuhnert, 1998	Germany	Retrospective multicenter study	46	pulse oximetry combined with cardiotocograph and fetal scalp pH	Apgar scores, cord gases, and transfer to the neonatal intensive care unit	Fetal arterial oxygen saturation less than 30% for more than 10 min correlates with low scalp pH values	[14]
Smits, 1989	The Netherlands)	Experimental study	14	Laser Doppler flowmetry	Scalp blood flow and transcutaneous pO_2_ during labor.	Local scalp blood flow modifies the partial pressure of O_2_ and is not a reliable parameter for monitoring fetal carotid arterial pO	[15]
Dupuis, 2018	France	Experimental descriptive study	-	fetal heart rate monitoring and blood gases analysis	heart rate, mean blood pressure, intra-amniotic pressure, pH, lactate pO_2_, pCO_2_, HCO_3_	Fetal pH normalizes between 20 and 30 min after a prolonged deceleration seen on FHR monitoring	[16]
Bligard, 2022	USA	retrospective cohort study	2081	fetal acidemia (umbilical artery pH < 7.2)	Neonatal death, encephalopathy, therapeutic hypothermia, seizures, intubation, and respiratory distress	Even a brief period of mild acidemia is associated with adverse neonatal outcomes	[24]
Frenken, 2022	the Netherlands	retrospective study	762	Fetal blood sampling because of pathological fetal heart rate tracings	Fetal hypoxia	Low fetal scalp pH values were significantly associated with contraction frequency (babies from women with more than 4 contractions/ ten minutes were more likely to develop hypoxia during labor)	[25]
Holzmann, 2015	Sweden	Prospective cohort study	1070	fetal scalp blood sampling	Lactate concentration, acid-base analysis	Repetitive fetal blood sampling is safe for the baby and more than half of the pregnant women will deliver vaginally.	[44]
Gilbert, 2021	France	retrospective monocentric cohort study	148	Fetal blood sampling and fetal scalp stimulation	Fetal heart rate and pH	Fetal scalp stimulation is a non-invasive alternative to blood sampling	[45]
Carbonne, 2016	Monaco	review	-	Fetal blood sampling	Fetal acidosis, fetal asphyxia and cerebral palsy	No well-designed, properly powered, randomized, control trial is available to compare cardiotocography with fetal blood sampling.	[5]
Simkovich, 1981	USA	Brief review	-	Fetal scalp pH	Fetal acidosis	A simplified pH electrode for continuous fetal scalp monitoring is needed	[30]
Cummings, 2018	UK	review	-	New types of physio-chemical sensors for lactate assessment	Fetal acidosis	A device less invasive for continuous monitoring of lactate levels is required.	[31]
Griffiths, 2022	USA	review	-	Fetal heart rate monitoring, fetal scalp blood sampling	chronic hypoxia, fetal distress	Scalp blood sampling is not a good method for assessment of the fetal distress because the skin of the fetal scalp undergoes catecholamine induced vasoconstriction in early stages of fetal distress	[33]
Antoine, 1982	USA	Review	-	Fetal heart rate, tissue pH and uterine contraction analysis	Fetal heart rate, tissue pH and uterine contraction analysis	Continuous tissues pH monitoring is a great adjutant to fetal heart monitoring for high-risk fetuses	[34]
Wagner, 1981	USA	Experimental study	9	Fetal heart rate monitoring and continuous scalp pH monitoring	Sudden decelerations and tissues pH level monitoring	pH measurement revealed for 5 cases that the decelerations were not a sign of fetal distress.	[35]
Lauersen, 1979	USA	Experimental study	40	Continuous fetal scalp tissue pH electrode	Acid-base equilibrium- fetal acidemia	Continuous fetal scalp tissue pH monitoring can be successfully used as clinical adjunct to fetal heart rate monitoring in cases where severe decelerations occur.	[36]
O’Dowd, 1987	UK	Experimental study	-	Novel fetal probe constructed with various hydrogen ion-selective polymer membrane	pH measuring of fetal scalp blood	The sensor based on TDDA liquid membrane is better suited for intermittent pH determinations	[37]
Rajala, 2021	Finland	prospective clinical study	187	fetal blood sampling of non-reassuring intrapartum CTG	Peripheral lactate concentration and pH	FBS lactate levels could not be used to predict fetal asphyxia	[38]
Bowler, 2014	Australia	retrospective cohort study	661	cord blood lactate concentration measured with a portable device	arterial lactate concentration, arterial pH and base excess	Cord blood lactate concentration is a good predictor of cord blood base excess and pH	[46]
Uchida, 2016	Japan	Experimental study	-	Newly–developed portable oximetry sensor attached to the medics’ finger	fetal head tissue oxygen saturation	The experiments on mice showed this device could be used to reliably monitor the oxygen saturation in the fetal scalp tissue.	[47]
Raghuraman, 2017	USA	Brief review (update)	-	Fetal heart rate patterns monitoring	Fetal acidemia	Category II fetal heart tracings cannot predict fetal acidemia	[48]
Dulay, 2021	Spain	Experimental study	-	Electrochemical multiparametric microsensors (O_2_ and pH assessment)	Fetal ischemia	Both sensors seemed feasible for future fetal monitoring applications since significant differences were observed for pH and pO_2_ parameters under normoxia and hypoxia.	[49]
Knupp, 2020	USA	review	-	Electronic fetal monitoring	fetal heart rate tracing	New emerging monitoring systems such as Monica Novii^®^ and Moyo could overcome the bias in interpretation of fetal heart rate tracings.	[50]
Steer, 2014	UK	Clinical opinion	-	Fetal blood sampling	Fetal acidemia	Acidosis is evaluated by measuring the pH	[20]
Prouheze, 2021	France	single-center retrospective study	268	fetal blood sampling	Scalp pH	For ˃25% of cases, the results were discordant between scalp pH and lactates	[51]
Chatterjee, 1984	USA	Experimental study	42	Fetal tissue pH using a glass electrode (Roche- Kontron)	Comparison between capillary pH and umbilical artery pH	The results of the fiberoptic tissue pH probe evaluated in vitro were promising.	[22]
Feduniw, 2022	Poland	retrospective analysis	395	Cardiotocography, blood sampling	Abnormal cardiotocography, Apgar score and pH	Abnormal cardiotocography in fetuses with congenital heart defects does not predict acidemia in these babies.	[27]

* The procedures we examined in this brief review were: electronic fetal monitoring (checking the condition of the baby heart rate), umbilical artery cord gas analysis (which provide information about a baby’s respiratory and metabolic status by analyzing the acid base equilibrium), auscultation (also checks the fetuses’ heart rate via a stethoscope or a Doppler transducer), fetal scalp blood sampling (testing the pH in the capillary blood of the fetus to assert the level of oxygenation), pulse oximetry (non-invasive means to check the oxygenation in the capillary tissue), laser Doppler flowmetry (used for blood perfusion assessment because it allows clinicians to discern the blood flow by transducing the moving red cell into an electric signal), fetal scalp stimulation (a firm digital pressure on the head of the fetus is required to check the acceleration of the heart rate and predict normal metabolic status) and test strip method (colorimetric sensing of the lactate concentration which is an indirect measure of pH) ** The main outcome we focused our attention on was fetal distress produced by fetal acidosis (defined as high amounts of lactic acid in a fetus’ blood). Fetal distress is a general condition that encompasses all the signs that the baby is not well because he does not receive enough oxygen. But we also included other measured outcomes such as fetal ischemia/hypoxia (hypoxia given by reduced oxygenation and decreased blood flow), cerebral palsy (weakness in the muscles produced by disruption in the oxygen levels of the fetus), Apgar score (scoring system assessing the Appearance, Pulse, Grimace response, Activity and Respiration of the baby 1 min and 5 min after birth), cesarean delivery and neonatal morbidities (encephalopathy, hypothermia, seizures, intubation, respiratory distress, transfer to neonatal intensive care unit).

## Data Availability

Not applicable.

## Appendix A

The queries we used for each scientific database are: “Foetal/fetal scalp blood sampling”, “fetal scalp pH”, “emergency cesarean delivery”, “Fetal tissue pH monitoring”, “electronic fetal monitoring” and “fetal sensors”. The databases were: ScienceDirect, Sage and PubMed.

## References

[1] Valenzuela C., Gregory E., Martin J. (2022). Decline in perinatal mortality in the United States, 2017–2019. NCHS Data Brief.

[2] Murray M., Huelsmann G., Koperski N. (2019). Essentials of Fetal and Uterine Monitoring.

[3] Osterman M. (2022). Changes in primary and repeat cesarean delivery: United States, 2016–2021. Vital Statistics Rapid Release.

[4] Faisant M., Fontecave-Jallon J., Genoux B., Rivet B., Dia N., Resendiz M., Riethmuller D., Equy V., Hoffmann P. (2022). Non-invasive fetal monitoring: Fetal Heart Rate multimodal estimation from abdominal electrocardiography and phonocardiography. J. Gynecol. Obstet. Hum. Reprod..

[5] Carbonne B., Pons K., Maisonneuve E. (2016). Foetal scalp blood sampling during labour for pH and lactate measurements. Best Pract. Res. Clin. Obstet. Gynaecol..

[6] Cahill A., Tuuli M., Stout M., Deych E., Shannon W., Macones G. (2016). Predicting normal pH with Intrapartum electronic fetal monitoring (EFM). Am. J. Obstet. Gynecol. Suppl. Jan..

[7] Haverkamp A., Orleans M., Langendoerfer S., McFee J., Murphy J., Thompson H.E. (1979). A controlled trial of the differential effects of intrapartum fetal monitoring. Am. J. Obstet. Gynecol..

[8] Nordstrom L., Ingemarsson I., Persson B., Shimojo N., Westgren M. (1994). Lactate in fetal scalp blood and umbilical artery blood measured during normal labor with a test strip method. Acta Obs. Gynecol. Scand..

[9] Westgren M., Kruger K., Ek S., Grunevald C., Kublickas M., Naka K., Wolff K., Persson B. (1998). Lactate compared with pH analysis at fetal scalp blood sampling: A prospective randomised study. Br. J. Obstet. Gynaecol..

[10] Martin A., Gaillard M., Miot S., Riethmuller D., Schaal J.P. (2003). Lactate measurements and acid-base balance in cord blood. J Gynecol. Obstet. Biol. Reprod..

[11] Valverde M., Puertas A., Lopez-Gallego M., Carrillo M., Aguilar A., Montoya F. (2011). Effectiveness of pulse oximetry versus fetal electrocardiography for the intrapartum evaluation of nonreassuring fetal heart rate. Eur. J. Obstet. Gynecol. Reprod. Biol..

[12] Goffinet F., Langer B., Carbonne B., Berkane N., Tardif D., le Goueff F., Laville M., Maillard F. (1997). Multicenter study on the clinical value of fetal pulse oximetry. Am. J. Obstet. Gynecol..

[13] Seelbach-Göbel B., Heupel M., Kühnert M., Butterwegge M. (1999). The prediction of fetal acidosis by means of intrapartum fetal pulse oximetry. Am. J. Obstet. Gynecol..

[14] Kuhnert M., Seelbach-Goebel B., Butterwegge M. (1998). Predictive agreement between the fetal arterial oxygen saturation and fetal scalp pH: Results of the German multicenter study. Am. J. Obstet. Gynecol..

[15] Smits T., Aarnoudse J., Zijlstra W. (1989). Fetal scalp blood flow as recorded by laser Doppler flowmetry and transcutaneous PO2 during labour. Early Hum. Dev..

[16] Dupuis H., Ghesquière L., Dejonckheere J., Aubry E., Sharma D., Storme D.P.L., Houfflin-Debarge V., Garabedian C. (2018). When should foetal pH measurements be performed after a prolonged deceleration? An experimental study in a fetal sheep model. Eur. J. Obstet. Gynecol. Reprod. Biol..

[17] R. The Ministry of Health and the Ministry of Education, ORDER nr. 1850/2019/3069/2020, Bucharest: Monitorul Oficial NR. 115 bis din 14 Februarie 2020, 11 December 2029. https://legislatie.just.ro/Public/DetaliiDocument/223062.

[18] Health M.O. (2019). Curriculum Preparation in the Specialty Obstetrics-Gynecology, Bucharest: Center for Human Resources in Public Health. https://lege5.ro/Gratuit/gm3dcnzugazq/ministerul-sanatatii-2019-curriculum-de-pregatire-in-specialitatea-obstetrica-ginecologie-anexa?dp=gmytaojsgq4demy.

[19] Chandraharan E. (2014). Fetal scalp blood sampling during labour: Is it a useful diagnostic test or a historical test that no longer has a place in modern clinical obstetrics?. BJOG. Int. J. Obstet. Gynaecol..

[20] Steer P. (2014). Fetal scalp blood sampling during labour: Is it a useful diagnostic test or a historical test which has no longer a place in modern clinical obstetrics. BJOG. Int. J. Obstet. Gynaecol..

[21] Qi X., Yang M., Ren W., Jia J., Wang J., Han G., Fan D. (2013). Find duplicates among the PubMed, EMBASE, and Cochrane Library Databases in systematic review. PLoS ONE.

[22] Chatterjee M., Hetzel F., Kaminetzky H. (1984). Fetal Tissue Phcontinuous Intrapartum Monitoring. Int. J. Gynaecol. Obstet..

[23] Da Silva Neto M.G., do Vale Madeiro J., Gomes D. (2022). On designing a biosignal-based fetal state assessment system: A systematic mapping study. Comput. Methods Programs Biomed..

[24] Bligard K., Cameo T., McCallum K., Rubin A., Rimsza R., Cahill A., Palanisamy A., Odibo A., Raghuraman N. (2022). The association of fetal acidemia with adverse neonatal outcomes at time of scheduled cesarean delivery. Am. J. Obstet. Gynecol..

[25] Frenken M., van der Woude D., Dieleman J., Oei S., van Laar J.H. (2022). The association between uterine contraction frequency and fetal scalp pH in women with suspicious or pathological fetal heart rate tracings: A retrospective study. Eur. J. Obstet. Gynecol..

[26] Simpson K., James D. (2008). Effects of oxytocin-induced uterine hyperstimulation during labor on fetal oxygen status and fetal heart rate patterns. Am. J. Obstet. Gynecol..

[27] Feduniw S., Muzyka-Placzyńska K., Kajdy A., Wrona M., Sys D., Szymkiewicz-Dangel J. (2022). Intrapartum cardiotocography in pregnancies with and without fetal CHD. J. Perinat Med..

[28] Alfirevic Z., Devane D., Gyte G., Cuthbert A. (2017). Continuous cardiotocography (CTG) as a form of electronic fetalmonitoring (EFM) for fetal assessment during labour. Cochrane Database Syst. Rev..

[29] Miller L., Miller D., Cypher R. (2022). Mosby’s^®^ Pocket Guide to Fetal Monitoring: A Multidisciplinary Approach.

[30] Simkovich J. (1981). Monitoring of fetal Scalp pH. (Symposium on Perinatal Diagnosis). Clin. Lab. Med..

[31] Cummins G., Kremer J., Bernassau A., Brown A., Bridle H., Schulze H., Bachmann T., Crichton M., Denison F., Desmulliez M. (2018). Sensors for Fetal Hypoxia and Metabolic Acidosis: A Review. Sensors.

[32] Bobrow C., Soothill P. (1999). Causes and consequences of fetal acidosis. Arch. Dis. Child. -Fetal Neonatal Ed..

[33] Griffiths K., Gupta N., Chandraharan E. (2022). Intrapartum fetal surveillance: A physiological approach. Obstet. Gynaecol. Reprod. Med..

[34] Antoine C., Silverman F., Young B. (1982). Current status of continuous fetal pH monitoring. Clin. Perinatol..

[35] Wagner P.G. (1981). Continuous fetal tissue pH monitoring-A preliminary experience. JOGN Nurs..

[36] Lauersen N.H., Miller F., Paul R. (1979). Continuous intrapartum monitoring of fetal scalp pH. Am. J. Obstet. Gynecol..

[37] O’Dowd M., Martin M.J., Wheble A., Gillmer M., Rolfet P. Ion-selective sensors for assessment of the fetus. Proceedings of the 27th Annual Scientific Meeting of the Biological Engineering Society.

[38] Rajala K., Mönkkönen A., Saarelainen H., Keski-Nisula L. (2021). Fetal lactate levels align with the stage of labour. Eur. J. Obstet. Gynecol. Reprod. Biol..

[39] Labrecque L., Caqueret A., Provençal M., Bujold E., Bédard M.-J. (2012). To foetal monitoring by measuring lactate scalp: Correlation of lactate and pH measured in arterial and venous cord blood. Clin. Biochem..

[40] Hutchison J., Mahdy H., Hutchison J. (2022). “Stages of Labor”, National Library of Medicine. https://www.ncbi.nlm.nih.gov/books/NBK544290/.

[41] Urang S., Davis L., Elsbery C.C., Kodowski M. (1993). Fetal Scalp Blood Sampling. J. Nurse-Midwifery.

[42] Williams K., Galerneau F. (2003). Intrapartum fetal heart rate patterns in the prediction of neonatal acidemia. Am. J. Obstet. Gynecol..

[43] Clark S., Paul R. (1985). Intrapartum fetal surveillance: The role of fetal scalp blood sampling. Am. J. Obstet. Gynecol..

[44] Holzmann, Wretler S., Cnattingius S., Nordström L. (2015). Neonatal outcome and delivery mode in labors with repetitive fetal scalp blood sampling. Eur. J. Obstet. Gynecol. Reprod. Biol..

[45] Gilbert M., Ghesquiere L., Drumez E., Subtil D., Fague V., Berveiller P., Garabedian C. (2021). How to reduce fetal scalp blood sampling? A retrospective study evaluating the diagnostic value of scalp stimulation to predict fetal wellbeing assessed by scalp blood sampling. Eur. J. Obstet. Gynecol. Reprod. Biol..

[46] Bowler T., Beckmann M. (2014). Comparing fetal scalp lactate and umbilical cord arterial blood gas values. Aust. N. Z. J. Obstet. Gynaecol..

[47] Uchida T., Kanayama N., Kawai K., Niwayama M. (2016). Craniofacial tissue oxygen saturation is associated with blood pH using an examiner’s finger-mounted tissue oximetry in mice. J. Biomed. Opt..

[48] Raghuraman N., Cahill A. (2017). Update on Fetal Monitoring—Overview of Approaches and Management of Category II Tracings. Obstet. Gynecol. Clin. N. Am..

[49] Dulay S., Rivas L., Pla L., Berdún S., Eixarch E., Gratacós E., Illa M., Mir M., Samitier J. (2021). Fetal ischemia monitoring with in vivo implanted electrochemical multiparametric microsensors. J. Biol. Eng..

[50] Knupp R., Andrews W., Tita A. (2020). The future of electronic fetal monitoring. Best Pract. Res. Clin. Obstet. Gynaecol..

[51] Prouhèze A., Girault A., Barrois M., Lepercq J., Goffinet F., le Ray C. (2021). Fetal scalp blood sampling: Do pH and lactates provide the same. J. Gynecol. Obstet. Hum. Reprod..

[52] Vavrinsky E., Esfahani N.E., Hausner M., Kuzma A., Rezo V., Donoval M., Kosnacova H. (2022). The Current State of Optical Sensors in Medical Wearables. Biosensors.

[53] Murray M. (2007). Antepartal and Intrapartal Fetal Monitoring.

